# Ciguatera Fish Poisoning in the Pacific Islands (1998 to 2008)

**DOI:** 10.1371/journal.pntd.0001416

**Published:** 2011-12-13

**Authors:** Mark P. Skinner, Tom D. Brewer, Ron Johnstone, Lora E. Fleming, Richard J. Lewis

**Affiliations:** 1 National Research Centre for Environmental Toxicology (Entox), The University of Queensland, Queensland, Australia; 2 ARC Centre of Excellence for Coral Reef Studies, James Cook University, Townsville, Queensland, Australia; 3 Coastal Ecosystems and Resource Management, School of Geography, Planning and Environmental Management and Centre for Marine Studies, The University of Queensland, St. Lucia, Queensland, Australia; 4 European Centre for Environment and Human Health, Peninsula College of Medicine, Truro, Cornwall, United Kingdom; 5 National Science Foundation (NSF)-National Institute of Environmental Health Sciences (NIEHS) Oceans and Human Health Center, Rosenstiel School of Marine and Atmospheric Sciences, University of Miami, Miami, Florida, United States of America; 6 Institute of Molecular Bioscience, The University of Queensland, St. Lucia, Queensland, Australia; Case Western Reserve University School of Medicine, United States of America

## Abstract

**Background:**

Ciguatera is a type of fish poisoning that occurs throughout the tropics, particularly in vulnerable island communities such as the developing Pacific Island Countries and Territories (PICTs). After consuming ciguatoxin-contaminated fish, people report a range of acute neurologic, gastrointestinal, and cardiac symptoms, with some experiencing chronic neurologic symptoms lasting weeks to months. Unfortunately, the true extent of illness and its impact on human communities and ecosystem health are still poorly understood.

**Methods:**

A questionnaire was emailed to the Health and Fisheries Authorities of the PICTs to quantify the extent of ciguatera. The data were analyzed using t-test, incidence rate ratios, ranked correlation, and regression analysis.

**Results:**

There were 39,677 reported cases from 17 PICTs, with a mean annual incidence of 194 cases per 100,000 people across the region from 1998–2008 compared to the reported annual incidence of 104/100,000 from 1973–1983. There has been a 60% increase in the annual incidence of ciguatera between the two time periods based on PICTs that reported for both time periods. Taking into account under-reporting, in the last 35 years an estimated 500,000 Pacific islanders might have suffered from ciguatera.

**Conclusions:**

This level of incidence exceeds prior ciguatera estimates locally and globally, and raises the status of ciguatera to an acute and chronic illness with major public health significance. To address this significant public health problem, which is expected to increase in parallel with environmental change, well-funded multidisciplinary research teams are needed to translate research advances into practical management solutions.

## Author Summary

Ciguatera fish poisoning occurs throughout the tropics. After consuming contaminated coral reef fish, people report a range of acute neurologic, gastrointestinal, and cardiac symptoms, with some experiencing chronic neurologic symptoms lasting weeks to months. Ciguatera is largely caused by toxins from benthic microalgae of the genera *Gambierdiscus* that are bioaccumulated in reef fish through the marine food chain. Unfortunately, the true extent of illness and its impact on human communities and ecosystems are still not well understood. Using data gathered from Health and Fisheries Authorities of the Pacific Island Countries and Territories we identified a 60% increase in the annual incidence of ciguatera from 1988–2008 to 1973–1983 and estimate over 500,000 Pacific islanders might have suffered from ciguatera in their lifetime. The incidence of ciguatera is expected to continue to rise in conjunction with continued reef degradation and global warming, with greatest impact likely to be experienced in the developing PICTs and potentially the archipelagoes of southeast Asia. Despite this threat, little funding is available for research that might lead to better management of the problem either locally, regionally or globally.

## Introduction

The developing Pacific Island Countries and Territories (PICTs) are under increasing threat from both acute and chronic diseases ranging from HIV/AIDS to obesity. In addition, people residing in PICTs are highly vulnerable to environmental impacts from the sea level rise and extreme weather events associated with global warming. Ciguatera is a prevalent tropical and subtropical disease that has been an under-appreciated cause of acute and chronic disease in island communities and might be increasing in incidence due to increasing vulnerabilities (i.e. poverty, global warming, eutrophication) in these populations [1]–[3].

Ciguatera is caused by the consumption of coral reef fish contaminated by ciguatoxin and related toxins from dinoflagellates (microalgae) and cyanobacteria [1], [2]. The ciguatoxin bioaccumulates up the food web, either directly from incidental uptake by herbivorous fish or indirectly by carnivorous fish [1]. After the consumption of coral reef fish contaminated with ciguatoxin, people experience potentially severe acute neurologic, gastrointestinal and cardiac symptoms as well as, in some cases, chronic neurologic symptoms lasting weeks to months [1], [2]. Ciguatera occurs globally, in coastal tropical waters, and is particularly prevalent across the PICTs. Cases of ciguatera have also been reported in temperate regions of the world due to travel and coral reef fish export. Ciguatera poisoning is often under-diagnosed and under-reported, with only 2 to 10% of cases reported to health authorities (Friedman et al 2008). Estimates of the incidence of ciguatera in Oceania have ranged from 0.5/10,000/year in Hawaii to 5,850/10,000/year in French Polynesia [2]. Fish is the staple protein source in many PICT communities, with many islands in the region suffering ongoing outbreaks of ciguatera leading to potentially significant impacts on large portions of the population of small island communities when toxic fish are consumed [3].

Dinoflagellates of the genera *Gambierdiscus*, that grow epiphytically on macro- and turf-algae on coral reefs, produce the ciguatoxins predominantly responsible for the disease known as ciguatera. Coral reef damage, or when algal growth is not controlled by herbivorous fish, provide increased potential habitat for *Gambierdiscus* growth that might increase the risk of ciguatera [4]. Despite extensive research, we know little about the ecology and the environmental factors that cause the blooms of the ciguatera caustive organisms, nor do we understand the role (if any) of other dinoflagellate genera including *Ostreopsis* (palytoxin producers) and *Prorocentrum* (okadaic acid and dinophysistoxins producers) or marine cyanobacteria [5]. Presently, ciguatoxin can only be detected in fish and *Gambierdiscus* in specialized labs, and diagnosis in humans is based almost exclusively on symptoms associated with the recent consumption of a potential ciguateric fish; factors that hamper its effective management and highlight important research needs [1], [2].

A number of factors have been associated with ciguatera cases and the presence of ciguatoxic dinoflagellates. Military activities causing coral reef damage in the Pacific, including from World War II, and nuclear test explosion programs, have been linked with outbreaks and changing incidence of ciguatera in some locations [6]. The prevalence of ciguatera in the South Pacific increases dramatically where average sea surface temperatures are at least 28 to 29°C [7]. Elevated sea surface temperatures associated with global warming are believed to already be exacerbating the extent and the range of ciguatera [8], . Reportedly, ciguatera occurrences are most prevalent in the warmest regions of the Caribbean, and all indigenous ciguatera cases have occurred where annual average temperatures are >25°C [4]. Nutrient enrichment and warming sea surface temperatures have been shown to stimulate *Gambierdiscus* growth which results in higher cell densities [10]. Also, benthic dinoflagellate species, including those of the genera *Gambierdiscus*, might have extended biogeographical ranges, induced by human activity. For example, benthic dinoflagellates are likely to be able to colonize previously unoccupied locations through transport in ship ballast [11]. Certain species of *Gambierdiscus* has now been found to be highly ciguatoxic compared to the other species [12], and blooms of these species are likely to contribute most to ciguatera risk.

Given changes in global climate patterns, increased degradation of coastal marine environments through coastal development and land run-off, and increased exploitation of coastal marine resources, the incidence of ciguatera cases is predicted to continue to increase in the future [4], [13]. Therefore, we hypothesized that ciguatera incidence is an increasing human health and ecological concern across the PICTs. To test this hypothesis, we report on changes in ciguatera incidence across the Pacific, and the social consequences of changing ciguatera incidence by comparing two 11 year periods of data: 1973–1983 vs 1998–2008.

## Methods

The Secretariat of the Pacific Community, the Institut Louis Malarde (Tahiti), the Institut Louis Pasteur (New Caledonia), and Institute for Research and Development (IRD) organized a Ciguatera workshop held in Noumea, October 2008. At this workshop, many island nation delegates declared a need for the ciguatera concern to be better addressed. To start to understand the current extent and nature of the ciguatera problem, we distributed a questionnaire to all PICTs (Supporting information S1). Ciguatera records used in this study are housed in each PICTs government health institution (Ministries and Departments of Health and Public Health).

### Questionnaire

To obtain the ciguatera records for the period of 1998 to 2008, we first contacted the Secretariat of the Pacific Community (SPC), Division of Fisheries, Aquaculture and Marine Ecosystems (FAME) to obtain the list of institutions responsible for maintaining ciguatera records within PICTs. We considered these repositories were comaparable to the data collection repositories used in the Lewis et al. study [14]. The questionnaire (Supporting Information S1) was sent by email to the institutions in October 2009. Updates on the returning of the questionnaires were sent to PICTs on four occasions over the period, and questionnaires were returned from the PICTs up until April 2010.

The questionnaire was developed by the co-authors in collaboration with the PICTs. The questionnaire included questions and definitions from prior ciguatera studies to provide consistency of data gathering and allow comparison across studies. The 3 key sections of the questionnaire collected information on: 1) Temporal incidence of ciguatera; 2) Environmental disturbance, to examine if coral reef condition and occurrence of coral bleaching and cyclones might influence ciguatera incidence (these data were considered purely speculative on the part of the respondent); and 3) Social consequences of ciguatera including changing diet and associated medical conditions, proactive and reactive management of ciguatera, and the desire for external assistance in response to ciguatera.

### Statistical analyses

#### 1) Temporal incidence of ciguatera

To determine whether the per capita incidence of ciguatera has increased significantly, as hypothesized, comparisons are made between the data from this study (1998–2008) and the work of Lewis [14] who reported on the epidemiology of ciguatera in the Pacific for the years 1973 to 1983. We tested for significant change in ciguatera incidence across PICTs by comparing mean annual incidence (per 100,000 people) within each PICT in each time period using a paired t-test, controlling for missing ciguatera values. We tested for overall change in ciguatera incidence using annual incidence (per 100,000 people) means across all PICTS for the two time periods using a independent-sample *t*-test and linear regression analysis, controlling for missing ciguatera values. All total incidence and incidence mean values were normalized prior to analysis using a natural log transformation. We also present the rates ratio (1998–2008 incidence/1973–1983 incidence), controlling for missing values.

#### 2) Environmental disturbance

We tested whether ciguatera incidence correlated with the incidence of cyclones or bleaching using independent-sample t-test. We also tested whether ciguatera incidence correlated with coral reef condition using spearman rank correlation. We used natural log transformed total incidence (per 100,000 people) within PICTs from 1998–2008 as our measure of incidence for all environmental disturbance analyses, controlling for missing ciguatera values.

#### 3) Social consequences of ciguatera

We tested whether the per capita incidence of ciguatera was associated with diet change, secondary medical problems, reactive management, proactive management, and perceived management benefit from additional support across the PICTs surveyed using independent-sample t-test, controlling for missing ciguatera values. A number of respondents did not complete this section of the questionnaire. Therefore, to ensure that non responses were not due to low or high ciguatera incidence, we also compared incidence rates between PICTs that responded and PICTs that did not, using independent-sample t-test.

## Results

Nearly all PICTs responded (17 or 85%), with half fully completing the ciguatera questionnaires. Whilst we contacted the health authorities for the ciguatera data (which were returned by the health authorities in all cases), other questions were left incomplete as they were not directly about the ciguatera health issue or were sent to the other government authorities to be fully completed.

### Temporal incidence of ciguatera

The reported cases for the recent 11 year period showed high levels of inter-year variability within and between PICTs. In Fiji, Kiribati and French Polynesia, more cases occurred at the start of the period. Annual reported cases peaked around the middle of the period at Cook Islands, Marshall Islands, Tokelau, Mariana's, and Hawaii. Reported cases in Vanuatu peaked towards the later part of the 11 year period and since 2005, Fiji experienced an increase in the number of ciguatera cases. Finally, Palau, Hawaii, Guam, Samoa, Wallis and Futuna, and Nauru all had relatively consistent incidence rates of under 5/100,000 (Table 1). Additional data relating to ciguatera incidence within PICT archipelagos are presented in Table 2.

**Table 1:** Ciguatera cases and mean annual incidence rates/100,000 by participating PICT: 1998–2008.

PICT	1998	1999	2000	2001	2002	2003	2004	2005	2006	2007	2008	Total	Population	Incidence
Cook Islands	215	156	138	133	183	227	469	421	258	245	242	2,687	17,000	1,436.90
French Polynesia	1,890	1,890	702	640	779	620	583	438	-	420	572	8,534	259,600	328.74
Fiji	1,754	2,827	1,932	1,715	1,100	559	547	428	617	772	1,004	13,255	837,000	143.97
Guam	1	7	7	5	5	4	0	4	4	2	3	42	165,000	2.31
State of Hawaii* †	-	41	37	59	68	64	35	27	25	18	31	405	1,250,000	3.24
Kiribati	361	467	675	524	463	184	63	77	46	64	259	3,183	92,500	312.83
North Marianas*	65	30	40	41	40	33	57	81	43	35	29	494	80,400	55.86
Marshall Islands*	-	118	112	178	482	171	233	195	245	178	210	2,122	51,000	416.08
New Caledonia	74	38	22	16	14	4	10	6	24	13	18	239	230,800	9.41
Nauru	0	0	0	0	0	0	0	0	0	0	0	0	10,065	0
Niue	0	0	0	0	20	0	1	2	15	1	11	50	1,500	303.03
Palau*	1	1	0	0	0	0	0	2	1	0	0	5	19,900	2.28
Samoa	1	4	3	2	3	5	2	-	-	-	-	20	180,741	1.58
Tokelau	58	30	39	20	43	20	18	7	14	16	14	279	1,609	1,576.36
Tonga	-	-	-	21	25	34	30	36	-	-	-	146	101,000	28.91
Tuvalu	0	0	12	0	0	0	36	1	34	4	2	89	9,600	84.28
Vanuatu	127	815	873	580	556	811	865	952	974	905	669	8,127	186,000	397.21
Wallis & Futuna	0	0	0	0	0	0	0	0	0	0	0	0	13,400	0
**Total**	**4,547**	**6,424**	**4,592**	**3,934**	**3,781**	**2,736**	**2,949**	**2,677**	**2,300**	**2,673**	**3,064**	**39,677**	**3,507,115**	**194.63**

*omitted from independent-sample t-test comparing annual means across time periods, and rates ratio calculation.

**†:** omitted from paired t-test comparing national means across time periods.

Note: Total incidence is the average annual incidence (per 100,000 people) for all PICTs for the entire 11 year period, controlling for missing values and excluding the state of Hawaii.

**Table 2:** Ciguatera cases and incidence rates/100,000 for selected islands of the participating PICTs: 1998–2008.

PICT	Island group	Ciguatera cases	Population	Mean incidence**
French Polynesia	Gambier	542	1,337	4,504
	Marquesas	2,636	8,632	3,393
	Tuamotu	3,590	15,510	2,571
	Australes	1,617	6,310	2,567
	Society	2,399	227,807	105
Vanuatu	Penama	1,855	26,600	699
	Sanma	1,933	36,100	488
	Malampa*	1,424	32,700	483
	Torba	329	8,800	341
	Shefa	1,969	54,400	329
	Tafea	537	29,000	169
Marshall Islands	Majuro	1,081	25,400	425
	Ralik chain	366	19,915	186
	Ratak chain	650	5,525	1,177
Kiribati	Southern Kiribati	413	1,519	2,502
	Central Kiribati	630	7,550	755
	Line Island	288	8,809	295
	Northern Kiribati	1,840	60,198	227
	Kanton (Phoenix)	13	41	2,927

*The numbers for Malampa were from 1998–2006.

**Incidence = cases/100,000 people.

**Figure 1:**
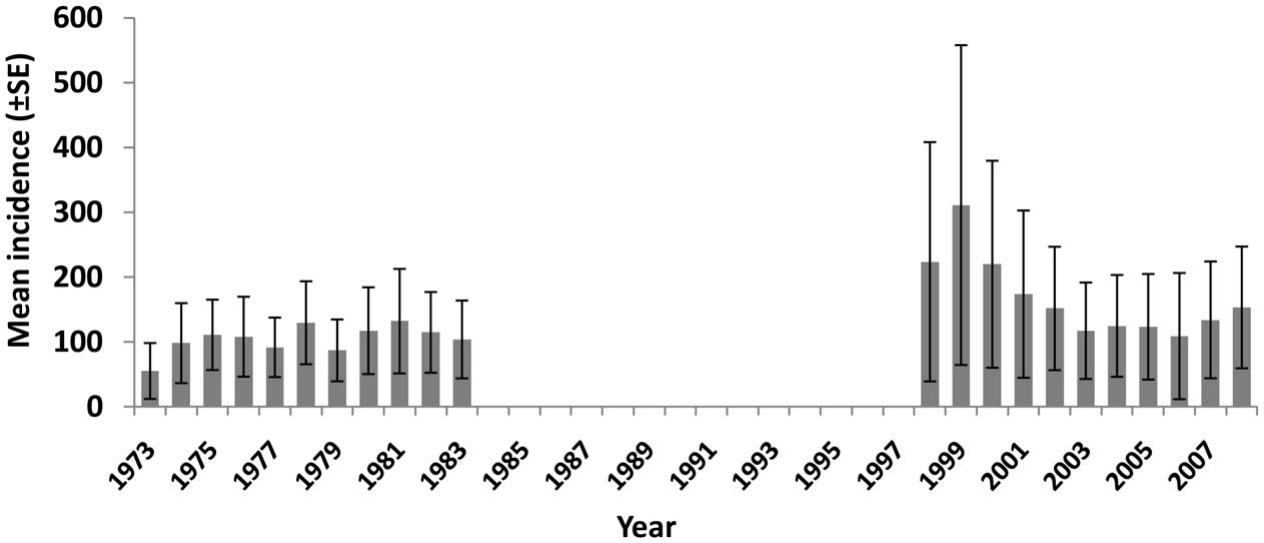
Annual incidence of ciguatera in the Pacific. Mean annual incidence (per 100,000 people) of ciguatera across Pacific Island Countries and Territories (PICT) from 1973–2008 are reported. Hawaii, North Marianas, Marshall Islands and Palau were omitted because comparable data was not available for both time periods.

Within the 35 year period (1973–2008), including the study by Lewis [14] and this study, there was a clear overall increase in CPF incidence; however, the results show inter-PICT incidence variability between the two time periods (Figure 1). Cook Islands, Vanuatu, Fiji, Tokelau, Marshall Islands, Niue, Tonga, and Palau all have increased ciguatera incidence (Table 3). Others have shown a decrease in ciguatera incidence in comparison to the other PICTs (such as Tuvalu and New Caledonia). From 1973–1983, only four nations demonstrated a ciguatera incidence over 300/100,000; now seven nations have an incidence over this value. Fiji now outranks French Polynesia as the nation with the highest number of ciguatera cases. Previously only four nations had over 2,000 ciguatera cases; recently six nations reported ciguatera cases over this value. Fiji, French Polynesia, Vanuatu, Kiribati, Cook Islands, and Tokelau all demonstrated an increase in the number of ciguatera cases; New Caledonia, Tuvalu and Guam showed a decrease in the number of cases.

**Table 3:** PICT rankings by ciguatera incidence/100,000: 1973–1983 vs 1998–2008.

PICT	1973–1983		1998–2008		
	Incidence	Rank	Incidence	Rank	Δ Rank
Cook Islands	2	16	1,453	2	>15
French Polynesia	565	2	344	3	<1
Fiji	17	11	144	8	>3
FSM	2	16	NR		NA
Guam	8	14	2	14	0
State of Hawaii	NR		3	18	NA
Kiribati	393	4	314	7	<3
Marshall Islands	282	5	416	4	>1
Nauru	11	13	0	16	<3
New Caledonia	148	6	10	12	<6
Niue	84	8	333	6	>2
North Mariana	130	7	56	10	<3
Palau	0	18	5	13	>4
Samoa	57	9	2	15	<6
Tokelau	995	1	1,554	1	0
Tonga	17	12	29	11	>1
Tuvalu	462	3	83	9	<6
Vanuatu	22	10	397	5	>5
Wallis & Futuna	5	15	0	16	<2

1973–1983 data (Lewis 1986); NR = No response.

Statistical analysis of temporal change in ciguatera incidence showed varied results. There was no statistically significant difference between 1973–1983 mean incidence and 1998–2008 mean incidence across PICTs (*p* = 0.949), comparing all PICTs (except the State of Hawaii which was not presented by Lewis [14]) using paired t-test (Table 4). However, there was a highly significant difference in mean incidence, between the two time periods when comparing mean incidence across years (*p* = 0.002), using independent-sample t-test. Linear regression analysis of annual mean incidence from 1973 to 2008 was also statistically significant (*p* = 0.005) despite high inter-year variability. The rate ratio (1998–2008 mean annual incidence/1973–1983 mean annual incidence) was 1.60; therefore, there was a 60% increase in the mean annual incidence from the earlier period to the more recent period. Hawaii, North Marianas, Marshall Islands, and Palau were omitted for the independent sample t-test, regression analysis and the rate ratio due to data limitations (Table 1).

**Table 4:** Change in the incidence of ciguatera: 1973–1983 vs 1998–2008.

	1973–1983 Mean (SD)	1998–2008 Mean (SD)	t	*p*-value (*r* ^2^)
PICT∧	167.3 (209.7)	300 (479.6)	−0.065	0.949
Year†	104.3 (21.6)	167.3 (61.5)	−3.617	0.002
Year‡	104.3 (21.6)	167.3 (61.5)	3.114	0.005 (0.33)

**∧:** paired sample t-test.

**†:** independent-sample t-test assuming equal variance.

**‡:** linear regression.

### Environmental disturbance

Of the 18 PICTs in this study, 11 reported on all (i.e. coral bleaching, cyclone incidence and perceived reef condition), whilst New Caledonia reported only on coral bleaching (Table 5). All three environmental disturbance types were positively related to ciguatera incidence. However, there was no statistically significant correlation between mean annual ciguatera incidence and occurrence of bleaching (*p* = 0.20), cyclone incidence (*p* = 0.17) or perceived coral reef condition (*p* = 0.57).

**Table 5:** Cyclones, coral bleaching and reef conditions reported by participating PICT: 1998–2008.

PICT	Bleaching	Cyclone	Reef Condition
Cook Islands	Yes	Yes	Poor
French Polynesia	-	-	-
Fiji	-	-	-
Guam	Yes	Yes	Poor
State of Hawaii	No	No	Good
Kiribati	No	No	Good
North Marianas	-	-	-
Marshall Islands	Yes	Yes	Good
North Caledonia	Yes	-	-
Nauru	-	-	-
Niue	Yes	Yes	Fair
Palau	No	No	Good
Samoa	Yes	Yes	Good
Tokelau	Yes	Yes	Fair
Tonga	Yes	Yes	Fair
Tuvalu	-	-	-
Vanuatu	-	-	-
Wallis & Futuna	No	No	Fair

### Social consequences of ciguatera

Responses to questions relating to the social consequences of ciguatera demonstrated that the incidence of ciguatera might be having a negative impact on PICT communities. Seven PICTs reported changes in diet as a result of ciguatera, whilst six PICTs reported that there was no change in diet as a result of ciguatera. Also, seven PICTs reported secondary medical problems (such as diabetes due to dietary changes) as a result of ciguatera. Five PICTs reported both a change in diet and secondary medical problems as a result of ciguatera. Seven PICTs reported taking reactive management measures (such as closure of fishing areas) to manage ciguatera outbreaks, whilst four PICTs reported taking no reactionary measures. Four PICTs reported that preventative management (such as catchment management) was occurring, whilst four PICTs reported that there was no preventative management. Eight PICTs reported that additional support would improve the management of ciguatera, whilst four reported that it would not.

There was a positive and marginally significant relationship between changing diet and per capita incidence of ciguatera (*p* = 0.06), and secondary medical problems and per capita incidence of ciguatera (*p* = 0.08) (Table 6). Neither reactive nor proactive management was correlated with per capita incidence of ciguatera. However, perceived improvement in management as a result of increased support was positively correlated with per capita incidence of ciguatera (*p* = 0.013). There was no significant difference (*p*≤0.05) in per capita incidence of ciguatera between nations that did and did not respond to questions on the social consequences of ciguatera.

**Table 6:** Relationships between per capita incidence of ciguatera and social consequences of ciguatera.

	No			Yes				
	N	Mean	St.Dev.	N	Mean	St.Dev.	t	*p*-value
Diet change	6	82.14	125.67	6	452.98	571.66	−2.123	0.060
Medical problems	5	90.83	133.53	7	554.38	669.26	−1.958	0.079
Reactive management	4	438.41	681.6	7	379.81	555.13	−0.371	0.719
Proactive management	5	444.54	649.49	4	546.24	619.78	−0.297	0.775
Additional support	4	8.2	13.84	8	525.11	624.19	−2.993	0.013

## Discussion

This study provides four important findings. First, as hypothesized, ciguatera incidence has increased significantly in the Pacific since the 1970s, but there is significant variability in incidence within PICTs since this time. Second, predicting causes of outbreaks and consequent elevated levels of ciguatera is difficult at the scale of this study, highlighting the need for further local-scale research and management action. Third, as reported earlier [3], ciguatera incidence continues to have significant negative effects on PICT societies, including dietary changes and associated medical problems (such as diabetes). Fourth, there has been inadequate response to date, yet there is acknowledgement from a number of PICTs that assistance would aid in the management of ciguatera. Such assistance could provide appropriate support and unified action might lead to solutions to a disease that could be considered an important cause of both acute and chronic illness in the Pacific.

Based on the results of this study compared to historical analyses, the overall incidence of ciguatera per 100,000 people appears to have increased significantly in the Pacific comparing 1973–1983 (mean104 cases/100,000 [14]) with 1998–2008 (mean194/100,000). There has been a 60% increase in the annual incidence of ciguatera between the two time periods based on PICTs that reported for both time periods (Figure 1). Two nations which exemplify the potential degree of change in incidence of ciguatera are the Cook Islands, where the incidence rose from 2/100,000 to 1,554/100,000 between the two time periods; and Tuvalu, where the incidence decreased from 462/100,000 people to 83/100,000 people. Furthermore, while it might appear that ciguatera incidence rates have subsequently fallen, they are still higher than the levels reported earlier by Lewis [14]. The non significant result from the paired *t*-test comparing within PICT ciguatera incidence for the two time periods suggests that there is significant variability of ciguatera incidence within PICTs through time. Therefore temporal change of incidence is difficult to predict at the PICT scale. However, the independent sample t-test and regression analysis revealed a regional increase in ciguatera incidence, highlighting the need for regional action.

Using the conservative estimate that the official reported ciguatera represents 20% of actual incidence [14], then the actual average overall incidence for the region would be 970/100,000 for 1998 to 2008. Others have estimated that only 5–10% of ciguatera cases are actually reported [2]. Across the region, using the reported mean values of actual cases for the three periods for which we have data (1,762 for 1973–1983; 2,844 for 1989–1992 (South Pacific Epidemiological and Health Information Services data); and 3,607 for 1998–2008 (current study)) and using a conservative under reporting rate of 80%, we estimate that since 1973 approximately 500,000 PICT inhabitants have had ciguatera.

It is possible that there might be a reporting bias in the data because of increased research and interest in ciguatera compared to the 1973–1983 time period. However, our data demonstrate high variability of ciguatera reporting from 1998–2008 across the PICTs. It is beyond the scope of this study to ascertain the effect of immigration and translocation of people to and from some of these PICTs, with different dietary habits than the local inhabitants, on ciguatera incidence. Given the variability in the change of incidence across the region demonstrated in this study, it is clear that the overall ciguatera trend cannot be extrapolated from data for a single PICT.

We elicited a relatively poor response rate from questions relating to coral bleaching, cyclones, and degraded reef conditions. Such environmental disturbance generally occurs at finer scales, so it might be appropriate to perform a more detailed field surveys in collaboration with environmental, fisheries, and meteorology agencies in the future to better understand such effects. However, despite the methodological limitations, we have shown that there is a trend for ciguatera incidence to be higher where bleaching, cyclones, and poor reef condition have been reported.

Stronger relationships were identified between ciguatera incidence and social impacts of ciguatera outbreaks. We found a marginally significant positive relationship between changing diet and the incidence of ciguatera, and associated medical problems and incidence of ciguatera. Such problems increase financial and social burdens on PICTs. Addressing the underlying causes of ciguatera outbreaks will reduce this burden, enabling PICT authorities to redistribute their limited resources to other priorities. Management action and prevention do not correlate with ciguatera incidence highlighting the lack of a unified and systematic approach for addressing ciguatera in the region. A clear desire for assistance exists within the PICTs that have high ciguatera incidence, suggesting that PICTs would be highly receptive to an external body aiding in enabling unified and systematic action. In addition to exploring new and better apporaches to detection and treatment, research is needed into the causes of ciguatera outbreaks, including environmental and anthropogenic parameters, to explain the hypotheses raised by this study.

### Limitations of study

It is possible that the unusual collaboration of the majority of the PICTs in this project might have contributed to the observed increased reporting of ciguatera (as well as other unidentified infrastructure changes), and thus a possible reporting bias for the more recent 1998–2008 data when compared with the 1973–83. However, these data represent a decade of reporting during a period of competing public health interests and lack of surveillance resources for ciguatera in the PICTs. As with all ciguatera studies where the case definition does not include active confirmation of ciguatoxin in the fish consumed, there is the possibility of the misclassification of reported cases; however, this situation has not changed from the 1970s [2].

It is also beyond the scope of this study to speculate on the causes of the high spatial and temporal variability of ciguatera [4]. This study, instead, aimed to demonstrate that ciguatera is still of major and possibly growing concern in the region. Addressing ciguatera will require significant investment in research and continuing education campaigns. If the suspected disturbances (including coral bleaching, cyclones, shipwrecks, and port facilities) are major causes of ciguatera outbreaks, then it is likely that the general temporal pattern of increased outbreaks will continue in the region, and be a far more expensive concern in the future, if our understanding of, and response to, ciguatera is not extensively improved.

### Conclusion

Despite over 50 years of ciguatera research in the Pacific, no comprehensive region-wide action has occurred to better manage ciguatera. Based on this study, an estimated 500,000 persons might have contracted ciguatera in the last 35 years, corresponding to a lifetime prevalence of 25%. It is remarkable that ciguatera has largely been ignored by the PICT national governments, with only two nations having an ongoing monitoring program and only one nation having a small unit devoted to researching ciguatera (Toxic Micro-algae Unit of the Institut Louis Malarde, French Polynesia). Given the rapidly changing physical environment (including global warming, extreme weather, and coral reef degradation) as well as the dependence of local populations upon fish for their physical and cultural survival, research into improved disease treatment and toxin detection, and a better understanding of the environmental factors contributing to ciguatera, are required to help reduce the likely growing adverse impacts of ciguatera.

## Supporting Information

Supporting Information S1 **Document and questionnaire sent to PICTs requesting questionnaire completion.**
(DOCX)
