# Supplementary material for: Ciguatera Fish Poisoning in the Pacific Islands (1998 to 2008)
Source: PLoS Negl Trop Dis. 2011 Dec 13;5(12):e1416. doi: 10.1371/journal.pntd.0001416 (PMC3236724; doi:10.1371/journal.pntd.0001416)
Supplement: Supporting Information S1 — Document and questionnaire sent to PICTs requesting questionnaire completion. (DOCX) [file pntd.0001416.s001.docx]

**Text S1. Document and questionnaire sent to PICTs requesting questionnaire completion.**

**Project: Analysis of Pacific Island Nations ciguatera fish poisoning cases 1998 to 2008** **Coordinators:** Mark Skinner (Entox, University of Queensland), Tom Brewer (JCU), & Richard Lewis (IMB, UQ).

**Invitation:**

Given global climate changes and increasing pressure on natural marine resources, ciguatera fish poisoning (CFP) caused by ciguatoxin and related toxins from dinoflagellates (microalgae) may increase and pose a greater threat to Pacific Island Nations and their way of life. At an SPC (ILM, ILP & IRD) led Ciguatera workshop held in October 2008, many island nation delegates declared a need for the Ciguatera problem to be addressed. Many islands have suffered ongoing outbreaks of ciguatera and others are experiencing it for the first time. The ability of toxic dinoflagellates to travel in ship ballast water, their invasiveness and global warming may increase the number of regions exposed to the risk of ciguatera.

The first step in addressing country needs in relation to ciguatera is to collect the ciguatera poisoning data from the most ciguatera prone regions in each Pacific Island country and present the data as a regional representation of the problem. This study will highlight the needs of those islands, with more emphasis needed by governments, NGO’s, regional organisations and fund providers, to put more monitoring and research into place. To assist management, so that well informed decisions and possible solutions can be made, to what is a very important environmental problem, impacting not only humans but all trophic levels of coral reef ecosystems and its biodiversity, on a local, national and regional basis.

Please fill in the attached questionnaire on behalf of your nation and ask your colleagues in other (Marine, Health, Environment, Ports) ministries to assist with your responses, where appropriate. Upon receiving this email and questionnaire, if you have not already participated, please let us know your intentions, so we can identify whether we can expect a response from your island nation and so that we don’t send further questionnaires. Please email (put ciguatera questionnaire in email topic box) your intent to participate (do not hesitate if you have any questions over its content) to: mark_skinner59@yahoo.com.au.

**On Completion of Questionnaire, please email back to:** mark_skinner59@yahoo.com.au

We will appreciate and acknowledge your efforts with what we see as one of the major environmental problems impacting Pacific Island nations.

Sincerely,

Mark Skinner, Tom Brewer & Richard Lewis

**Ciguatera in Pacific Island Nations, 1998 to 2007 Questionnaire**

Country: __________________________________________________________

Your Name: _______________________________________________________

Your Institution: ____________________________________________________

**Q1.** All reported ciguatera cases by nation (total) and region (worst 5 Islands or Regional Island groups, R1–R5, please place island or group name in top row)

| **Year** | **Total Number** | **R1** | **R2** | **R3** | **R4** | **R5** |
| --- | --- | --- | --- | --- | --- | --- |
| **1998** |  |  |  |  |  |  |
| **1999** |  |  |  |  |  |  |
| **2000** |  |  |  |  |  |  |
| **2001** |  |  |  |  |  |  |
| **2002** |  |  |  |  |  |  |
| **2003** |  |  |  |  |  |  |
| **2004** |  |  |  |  |  |  |
| **2005** |  |  |  |  |  |  |
| **2006** |  |  |  |  |  |  |
| **2007** |  |  |  |  |  |  |
| **2008** |  |  |  |  |  |  |

**Q2**. Nation population at last census (year of census) ___________________(_______)

**Q3**. Population of each island/ island group as reported in table 1 above?

R1. ___________ R2. ___________ R3. ___________R4.___________ R5._________

**Q4**. Year of first CFP cases since 1930. Earliest for the whole Nation: __________

R1. ___________ R2. ___________ R3. ___________ R4. ________ R5. _________

**Q5**. Cyclones and reef in the last 10 years (please place an X)

| **Island/group** | **Cyclones** | **Coral bleaching** | **Reef condition** |
| --- | --- | --- | --- |
| **Region 1** | Yes No | Yes No | Good Fair Poor |
| **Region 2** | Yes No | Yes No | Good Fair Poor |
| **Region 3** | Yes No | Yes No | Good Fair Poor |
| **Region 4** | Yes No | Yes No | Good Fair Poor |
| **Region 5** | Yes No | Yes No | Good Fair Poor |

**Q6.** Has ciguatera resulted in changes in diet away from reef fishes?: Yes No

**Q7**. Has ciguatera added to other medical problems (e.g. obesity, diabetes)?: Yes No

**Q8.** Have any actions been taken (e.g. closing off areas to fishing, marine park zoning, extra medical attention etc) on each of the islands/groups listed in the first table, to manage CFP outbreaks (please place an X)?

R1: Yes No R2: Yes No R3: Yes No R4: Yes No R5: Yes No

If YES, please indicate measures employed: ______________________________________

**Q9.** Is anything happening to reduce ciguatera (e.g. preventing construction of infrastructure of tourism and development, or catchment management):

R1: Yes No ? R2: Yes No ? R3: Yes No ? R4: Yes No ? R5: Yes No ?

If YES, please indicate measures employed: ______________________________________

**Q10.** Please state any major international shipping movements to any of the regions and any associated wharf or reef changes (i.e. harbor improvements, passage dredging or widening) over the last 10 years?

| **Island / island group** | **Vessel type, size (length)** | **Frequency of visits** | **Infrastructure changes (date)** |
| --- | --- | --- | --- |
| R1 |  |  |  |
| R2 |  |  |  |
| R3 |  |  |  |
| R4 |  |  |  |
| R5 |  |  |  |

**Q11**. For islands/ island groups with inhabitants and doctors/medical posts, **but no recorded cases of ciguatera**, please state region/island, major shipping movements, and any major changes to the wharf/ reef infrastructure of the island? (Please give an overview).

| **Island /region group name** | **Vessel type, size (length)** | **Frequency of visits** | **Infrastructure changes** |
| --- | --- | --- | --- |
|  |  |  |  |

**Q12.** Please indicate the first international shipping vessel arrivals (either ocean going, haulage, carrying containers, fishing trawlers, tourist cruise ships or military vessels etc) to visit each of the island/groups as listed in table 1 above.

R1.____________ R2.____________R3. ___________R4.___________R5._________

**Q13.** Please provide any dates (year), for the first large shipwrecks (either, ocean going, carrying containers, fishing trawlers, tourist cruise ships or military vessels etc) that have occurred in the island/groups as listed in table 1 above.

R1.____________R2.____________R3. ___________R4.___________ R5.__________

**Q14.** Please provide any dates (year), where there been any most recent wrecks, in the last ten years, on any of the islands/ island groups that maybe related to new outbreaks on any of the islands/groups listed in table 1 above?

R1.____________ R2.____________R3. ___________R4.___________ R5._________

**Q15.** Please provide any dates, for **the first** major shipping installations, passage widening or harbor infrastructure AND major land infrastructures (such as runway construction) that have occurred in the islands as listed in Q1.

R1.____________ R2.____________R3. ___________R4.___________ R5.__________

**Q16.** Please indicate fish most often implicated in ciguatera(e.g. red bass) by region from Q1.

R1.____________ R2.__________R3. ___________R4.___________R5.___________

**Q17.** Would additional help or support improve the management of ciguatera in your nation?

Yes No ? Anything to add?________________________________________________

Please list the key persons that have helped you compile the results for this questionnaire that should be acknowledged in the publication of this data. If you don’t wish to answer all questions we will accept incomplete questionnaires.

________________________________________________________________________

Thank you for completing this questionnaire and please return to: mark_skinner59@yahoo.com.au
